# Activation of G protein coupled estrogen receptor prevents chemotherapy-induced intestinal mucositis by inhibiting the DNA damage in crypt cell in an extracellular signal-regulated kinase 1- and 2- dependent manner

**DOI:** 10.1038/s41419-021-04325-z

**Published:** 2021-10-30

**Authors:** Guanyu Chen, Honghui Zeng, Xinyun Li, Jianbo Liu, Zhao Li, Runze Xu, Yuntao Ma, Chuanyong Liu, Bing Xue

**Affiliations:** 1 Department of Physiology and Pathophysiology, School of basic medical science, Cheeloo College of Medicine, Shandong University, Jinan, China; 2 The State and Shandong Province Joint Key Laboratory of Translational Cardiovascular Medicine, Qilu Hospital, Shandong University, Jinan, China; 3 Second Clinical Medical College, Lanzhou University, Lanzhou, China

**Keywords:** Pharmacology, Gastrointestinal diseases

## Abstract

Chemotherapy-induced intestinal mucositis (CIM) is a common adverse reaction to antineoplastic treatment with few appropriate, specific interventions. We aimed to identify the role of the G protein coupled estrogen receptor (GPER) in CIM and its mechanism. Adult male C57BL/6 mice were intraperitoneally injected with 5-fluorouracil to establish the CIM model. The selective GPER agonist G-1 significantly inhibited weight loss and histological damage in CIM mice and restored mucosal barrier dysfunction, including improving the expression of ZO-1, increasing the number of goblet cells, and decreasing mucosal permeability. Moreover, G-1 treatment did not alter the antitumor effect of 5-fluorouracil. In the CIM model, G-1 therapy reduced the expression of proapoptotic protein and cyclin D1 and cyclin B1, reversed the changes in the number of TUNEL^+^ cells, Ki67^+^ and bromodeoxyuridine^+^ cells in crypts. The selective GPER antagonist G15 eliminated all of the above effects caused by G-1 on CIM, and application of G15 alone increased the severity of CIM. GPER was predominantly expressed in ileal crypts, and G-1 inhibited the DNA damage induced by 5-fluorouracil in vivo and vitro, as confirmed by the decrease in the number of γH2AX^+^ cells in the crypts and the comet assay results. Referring to the data from GEO dataset we verified GPER activation restored ERK1/2 activity in CIM and 5-fluorouracil-treated IEC-6 cells. Once the effects of G-1 on ERK1/2 activity were abolished with the ERK1/2 inhibitor PD0325901, the effects of G-1 on DNA damage both in vivo and in vitro were eliminated. Correspondingly, all of the manifestations of G-1 protection against CIM were inhibited by PD0325901, such as body weight and histological changes, the mucosal barrier, the apoptosis and proliferation of crypt cells. In conclusion, GPER activation prevents CIM by inhibiting crypt cell DNA damage in an ERK1/2-dependent manner, suggesting GPER might be a target preventing CIM.

## Introduction

Chemotherapy-induced intestinal mucositis (CIM) is a common adverse reaction to antineoplastic treatment and the predominant reason for the poor survival and reduced quality of life of tumor patients [1]. CIM is characterized by structural, functional and immunological changes in the mucous membranes, mainly concentrated in small intestine and oral cavity [2]. Multiple factors are involved in the pathogenesis of CIM, such as cell apoptosis, proliferation inhibition, oxidative stress and inflammation [3, 4]. However, to date, few applicable and specific interventions are available for CIM [5].

Chemotherapeutic drugs targeting tumor cells elicit their antineoplastic effects by interfering with DNA replication to inhibit cell division, induce cell cycle arrest and apoptosis [6, 7]. However, antineoplastic drugs also indiscriminately target other rapidly proliferating cells, such as intestinal crypt cells [8, 9], including intestinal stem cells (ISCs) and their progeny transit amplifying cells (TACs). ISCs undergo asymmetric division to self-renew or give rise to rapidly proliferating TACs, which then differentiate into all epithelial lineages [10]. Intestinal crypt cells call upon clonogenic cells to replenish damaged sections and sustain the integrity of the intestinal barrier, which is indispensable for radiotherapy induced intestinal regeneration [11]. Crypt cell damage associated with DNA damage, such as apoptosis and proliferation inhibition, is one of the key mechanisms of CIM [8, 12–15]. Enhancing the survival of crypt cells following chemotherapy should be a potential effective treatment for CIM.

A previous report showed that hormone therapy consisting of estrogen-progesterone significantly reduced the required transfusion frequency in bleeding radiogenic colitis [16]. Estrogen plays an important role in maintaining the gastrointestinal mucosal barrier [17, 18] and its role is achieved through the activation of estrogen receptors, including the estrogen nuclear receptor and membrane receptor. G protein coupled estrogen receptor (GPER) is an important membranous estrogen receptor involved in the regulation of cell proliferation, apoptosis and migration, the immune response, metabolism, neural facilitation, and so on [19, 20]. Activation of GPER was related to the regulation of proliferation of cells with stem cell properties [21, 22] and its specific agonist G-1 protected epidermal stem cells against ultraviolet B-induced injury [23]. GPER was expressed in the gut of humans and other mammals and its activation was associated with colonic motility regulation, visceral pain, and inflammatory bowel disease [24–27]. Our group found the activation of GPER expressed in intestinal crypts inhibited apoptosis and protected the proliferation of crypt cells during intestinal ischemia/reperfusion injury and colitis [28, 29]. These results led us to wonder whether GPER plays a role in CIM by acting on crypt cells in the small intestine, which has not been reported so far. Therefore, in the present research we established a CIM model with 5-fluorouracil (5-FU) to investigate the role of GPER in CIM and focused on crypt cells to explore the mechanism.

## Materials and methods

### Animals

Six- to eight-week-old male C57BL/6 mice weighing 18–25 g were purchased from Charles River Laboratories (Beijing, China). Animals were reared in a room with a controlled temperature (25 ± 2 °C) and constant humidity (50% ± 5%) on a 12 h/12 h’ light/dark cycle. Animals had free access to water and food during the experiments. Sample size selection for the animal experiments was carried out per the preliminary experiments as well as similar previously reported experiments. All animal experiments were approved by the Medical Ethics Committee for Experimental Animals, Medical School, Shandong University, China.

### Establishment of the CIM model and experimental design

The CIM model was established by intraperitoneal injection of 5-FU (30 mg/kg/day) for 5 consecutive days [30]. The selective GPER agonist G-1 (0.03 mg/kg) and/or selective GPER antagonist G15 (0.3 mg/kg) were administered intraperitoneally together with 5-FU to detect the role of GPER in CIM. Mice were randomly divided into one of four subgroups or one of three subgroups, namely control group (saline), 5-FU vehicle group, 5-FU + G-1 group, 5-FU + G-1 + G15 group or control group, 5-FU vehicle group and 5-FU + G15 group. Preliminary experiments showed that either G-1 or G15 administration for 5 days did not affect the ileal histological features or body weight of untreated mice (data not shown). To observe whether ERK1/2 was involved in the effects of GPRE on CIM, the selective ERK1/2 inhibitor PD0325901 (5 mg/kg/day) was intraperitoneally injected into the 5-FU induced CIM model mice together with or without G-1. C57BL/6 mice were randomly assigned to one of the following groups: the 5-FU vehicle group, 5-FU + PD0325901 group, 5-FU + G-1 group or 5-FU + G-1 + PD0325901 group. All drugs were injected at 9 am every day for 5 consecutive days. The body weights of the mice were monitored daily from day 0 to day 5 and reported as a percentage change compared to their weight before treatment. Mice were sacrificed on the morning of day 5 and each ileum was separated for the following experiments.

We established another CIM model with cisplatin by referencing the literature with slight modifications according to our preliminary experiments [8, 12]. Cisplatin (5 mg/kg) was injected intraperitoneally on day 0 and day 2. G-1 (0.03 mg/kg/day) was administered for 5 consecutive days in cisplatin-induced CIM model mice. Weight changes were observed daily from day 0 to day 5, and the mice were sacrificed on day 5 for histological testing.

### Tumor-xenograft model

A total of 4 × 10^6^ LL/2 (Lewis lung carcinoma, LLC) cells (Nanjing Kebai Biological Technology Co., Ltd., China) were injected into the dorsal flanks of male C57BL/6 mice (18–20 g, 5–6 weeks old), which were a gift from Dr. Yanli Liu (Shandong Cancer Hospital Affiliated to Shandong University, China). When the size of the tumor xenograft reached approximately 100 mm^3^, the mice were randomly assigned to one of 3 groups: saline (control), 5-FU vehicle group, and 5-FU + G-1 group. Saline or drugs were intraperitoneally injected for 8 consecutive days. The volumes of the tumors were measured with digital calipers every day and calculated according to the formula: 0.5 × (length × width^2^).

### Hematoxylin and eosin (H&E) staining

Isolated segments from the ileum (2 cm each) were cleaned with cold PBS and fixed with 4% paraformaldehyde overnight, followed by dehydration in an gradient series of ethanol solutions, embedding in paraffin and sectioning into 4 μm for H&E staining. The degree of mucosal injury was microscopically measured and quantitatively analyzed in accordance with Chiu’s scoring system [31]. Briefly, 0: normal mucosal villi; 1: subepithelial spaces at the tip of villi; 2: extension of subepithelial detachments with moderate epithelial lifting; 3: large subepithelial detachments and extensive epithelial lifting with occasional denuded villi tips; 4: denuded villi with dilated capillaries; 5: lamina propria disintegration, ulceration, and hemorrhage. The crypt damage was scored under Kristen’s system [32]. 0: None; 1: Basal 1⁄3 damaged; 2: Basal 2⁄3 damaged; 3: Crypts lost, surface epithelium present; 4: Crypts and surface epithelium lost. At least 20 villi and crypts were measured on each slide and the mean value was calculated by two experienced observers blinded to the treatment.

### In vivo intestinal permeability measurement

To assess intestinal barrier function in vivo intestinal permeability was evaluated by the concentration of fluorescein isothiocyanate (FITC) -dextran (molecular weight, 4000 Da; Sigma–Aldrich, Saint Louis, MO, USA) in the blood. The mice were fasted overnight and gavaged with FITC-dextran (400 mg/kg, dissolved in 0.1 mL PBS) 4 h before sacrifice. Blood was obtained from the vena ophthalmica after anesthesia with isoflurane followed by centrifugation to obtain hemolysis-free serum. The intensity of the fluorescence was measured with a microplate reader (Molecular Devices, Silicon Valley, USA) at an excitation wavelength of 488 nm and an emission wavelength of 520 nm. The concentration of FITC-dextran was calculated based on a standard curve reflecting the relationship between the FITC-dextran concentration and fluorescence intensity.

### Immunohistochemistry (IHC)

The ileal paraffin slides were dewaxed with xylene, rehydrated in a gradient series of ethanol solutions, and heated to 120 °C in 10 mM citrate buffer for 30 min. Endogenous peroxidase activity was diminished by incubation with 3% H_2_O_2_ at room temperature for 30 min. Goat serum (ZSGB-BIO, China) was used to block the sections at 37°C for 30 min after rinsing with PBS. The sections were then incubated with mouse anti-bromodeoxyuridine (BrdU) antibody (1:300; 66241-1-Ig, Proteintech, USA), rabbit anti-Ki67 antibody (1:300; #12202, Cell signaling Technology, USA), rabbit anti-mucin-2 antibody (1:200; SC-515032, Santa Cruz Biotechnology, USA) or rabbit anti-Phospho-Histone H2A.X(Ser139) antibody (1:400; #9718, Cell signaling Technology, USA) at 4 °C overnight. The slides were incubated with a biotin-labeled secondary antibody (ZSGB-BIO) at 37 °C for 1 h and labeled with streptomyces avidin peroxidase (ZSGB-BIO), followed by treatment with a 3,3’-diaminobenzidine-tetrahydrochloride kit (ZSGB-BIO) for visualization. Hematoxylin was used to counterstain the nuclei. Measurements were taken by two experienced observers blinded to the experimental protocol.

### Immunofluorescence (IF)

After dewaxing, rehydration and antigen-repair, the slides were incubated with Triton-X-100 for 10 min at room temperature and blocked with goat serum (ZSGB-BIO) for 30 min at 37 °C. The sections were incubated with the primary antibody rabbit anti-G-protein coupled receptor 30 (1:50; GTX107748, GeneTex, USA), rabbit anti-Phospho-p44/42 MAPK (ERK1/2) (Thr202/Tyr204) (1:200; #4370, Cell Signaling Technology) or rabbit anti-ZO-1 (1:50; 21773-1-AP, Proteintech) at 4°C overnight. The slides were incubated with Rhodamine (TRITC)-conjugated Goat Anti-Rabbit IgG (H + L) (1:50; SA00007-2, Proteintech) or CoraLite488 conjugated Goat Anti-Rabbit IgG (H + L) (1:50; SA00013-2, Proteintech) as secondary antibody in a humid dark box at 37 °C for 60 min. 4’6-diamidino-2-phenylindole (DAPI) was used to label the nuclei. After the preparation against quenching, the slides were observed under fluorescence microscope (Nikon, Japan).

### 5-Bromo-2’-deoxyuridine (BrdU) incorporation

Mice were intraperitoneally injected with BrdU (50 μg/g) 2 h before sacrifice [33]. Each ileum was removed, embedded in paraffin and sliced, and then the slides were incubated with mouse anti-BrdU antibody following the IHC protocol described above.

### Protein isolation and western blot analyses

The protein was isolated from ileum tissues or cells [29] and the protein concentration was measured using a Bicinchoninic Acid Protein Assay kit (Beyotime Institute of Biotechnology, China). Protein samples were separated by sodium dodecyl sulfate–polyacrylamide gel electrophoresis and transferred to polyvinylidene difluoride (PVDF) membrane (Millipore, USA). The membrane was blocked with 5% nonfat dry milk for 2 h at room temperature and then was incubated at 4 °C overnight with rabbit anti-Cyclin D1 antibody (1:10000; ab134175, abcam, UK), rabbit anti-Cyclin B1 antibody (1:2000; ab181593, abcam), rabbit anti-ERK1 (pT202/pY204) + ERK2 (pT185/pY187) antibody (1:10000; ab76299, abcam), rabbit anti-ERK1 + ERK2 antibody (1:10000; ab184699, abcam), mouse anti-Phospho-SAPK/JNK (Thr 183/Tyr 185) antibody (1:2000; #9255, Cell Signaling Technology), mouse anti-SAPK/JNK antibody (1:1000; #9252, Cell Signaling Technology), rabbit anti-Phospho-p38 MAPK (Thr180/Tyr182) antibody (1:1000; #9211, Cell Signaling Technology), rabbit anti-p38 MAPK antibody (1:1000; #9212, Cell Signaling Technology), rabbit anti-ZO-1 antibody (1:1000; 21773-1-AP, Proteintech), rabbit anti-Cleaved Caspase-3 (Asp 175) (1:1000; #9661, Cell Signaling Technology), rabbit anti-Caspase-3 antibody (1:1000; 19677-1-AP, Proteintech), rabbit anti-Caspase-3 antibody (1:1000; #9662, Cell Signaling Technology), rabbit anti-Bcl-2 antibody (1:1000; 12789-1-AP, Proteintech), rabbit anti-Bax antibody (1:5000; 50599-2-Ig, Proteintech) or mouse anti-beta-actin antibody (1:5000; 66009-1-Ig, Proteintech). Blots were washed three times in TBST for 10 min and incubated with the HRP-conjugated goat anti-rabbit IgG (1:5000; SA00001-2, Proteintech) or peroxidase-conjugated goat anti-mouse IgG (1:5000; SA00001-1, Proteintech) at room temperature. After being washed in TBST, the blots were covered with BeyoECL PLUS (Beyotime Institute of Biotechnology, China) to visualize, and quantified with ChemiDoc XRS system and mage Lab Software (Bio-Rad, Hercules, USA).

### Terminal deoxynucleotidyl transferase-mediated dUTP nick-end labeling (TUNEL) assay

The TUNEL assay was conducted with an In Situ Cell Death Detection kit (Roche) to detect epithelial cell apoptosis. The paraffin slides were dewaxed, rehydrated and incubated with 20 μg/ml proteinase K for 15 min at room temperature. The slides were then incubated with TUNEL reaction mixture at 37 °C for 1 h. Nuclei were counterstained with DAPI. The slides were observed under fluorescence microscope (Nikon, Japan), and the number of TUNEL^+^ cells per crypt were counted by two experienced observers blinded to the experimental protocol.

### Cell culture and treatment in vitro

IEC-6 cells are intestinal epithelial cell line with undifferentiated characteristics of intestinal crypt cells and were purchased from American Type Culture Collection. Cells were cultured in 1640 medium with 10% fetal bovine serum (FBS), 100 μg/mL streptomycin and 100 unit/mL penicillin in humidified air containing 5% CO_2_ at 37 °C. First, cells were cultured in 6-well plates at a density of 5–10 × 10^5^/well for 24 h, after which the non-adherent cells were removed. Adherent cells were cultured with medium without FBS for 24 h to eliminate intrinsic ERK1/2 phosphorylation. The cells were then incubated with 0.5% FBS and stimulated with 10^−4 ^mol/L 5-FU for 48 h or 96 h with or without G-1 (10^−7 ^mol/L). The selective ERK1/2 inhibitor PD0325901 (5 × 10^−3^ mol/L) was administrated to block ERK1/2 phosphorylation. The final DMSO concentration in each group was 0.001% in PBS, so the control group was treated with 0.001% DMSO.

### Comet assay

This experiment was conducted with a Comet Assay reagent kit, purchased from Trevigen (Gaithersburg, MD, USA). Plates were washed with PBS to remove non-adherent cells and rinsed with the kit solution (0.25% trypsin, 0.53 mM EDTA) until all of the cells detached. The cell suspension was collected and centrifuged. The supernatant was removed and PBS was added to control the cell density. Cells (1 × 10^5^ /mL) were combined with molten LMAgarose at 37°C at a volume ratio of 1: 10. 50 μL of this mixture was quickly pipetted onto the comet slides. The slides were placed at 4 °C in the dark with no humidity for 15 min and then with high humidity for 30 min. The slides were immersed in lysis solution for 30 min and unwinding solution for 20 min at 4 °C in the dark. Next, the slides were subjected to electrophoresis at 21 volts for 30 min. SYBR solution was used to visualize the DNA by fluorescence microscopy. The images (100× magnification) were analyzed by CASP to obtain the tail length, tail DNA percentage, tail moment and olive tail moment values.

### GEO dataset and gene set variation analyses (GSVA)

We obtained gene expression data from GEO (www.ncbi.nlm.nih.gov/gds). We included the series based on the following criteria: key words: “small intestine” and “chemotherapy”; tissue from the small intestine; sample replication ≥ 3; and expression profile by array. One GEO dataset (GSE56426) of intestinal tissue was found and downloaded. The R package GSVA was applied to analyze the enrichment score of functions in each sample, and the R package limma was used to filter the significant differential functions related to pathways, the cell cycle and DNA damage. The threshold for difference analysis was set at *p* < 0.05.

### Drugs and chemicals

5-FU was bought from sigma-aldrich (St. Louis, MO, USA). G-1 and G15 were bought from ApexBio (USA) and Cayman Chemical (Ann arbor, USA), respectively. PD0325901 was bought from MedChemExpress (Monmouth Junction, USA). Cisplatin was purchased from Sigma.

### Statistical analysis

The data were shown as mean ± SEM. The comparisons between multi-groups were presented using the one-way analysis of variance followed by Newman-Keuls test. *P* < 0.05 was considered statistically significant. Data were analyzed using GraphPad Prism version 5.0.

## Results

### GPER activation inhibited the severity of CIM induced by 5-FU without affecting the antitumor effect of 5 -FU

**Fig. 1:**
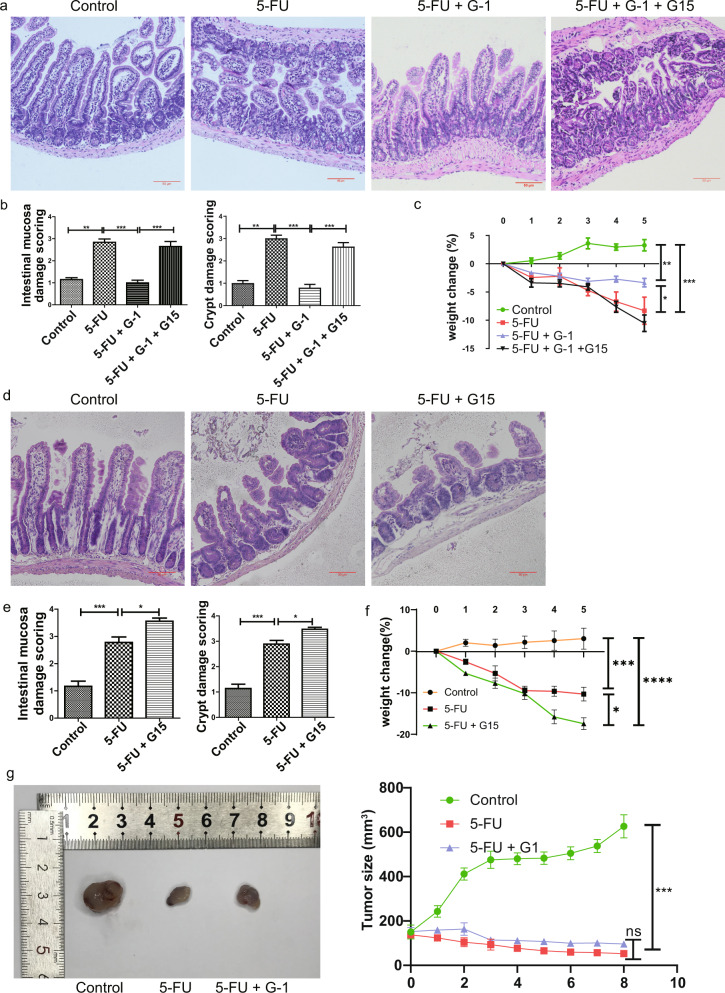
Effect of GPER on body weight and ileum histopathological damage in 5-FU induced CIM and on anti-tumor effect of 5-FU. CIM model was induced by i.p. injection 5-FU (30 mg/kg/day) for 5 days. The control group was intraperitoneally injected with the same amount of saline. Selective GPER agonist G-1 (0.03 mg/kg/day), selective GPER blocker G15 (0.3 mg/kg/day) were administrated intraperitoneally together with 5-FU. G-1 was administrated alone or with G15 together in the CIM model to test the effect of GPER activation. G15 was used alone to test the effect of endogenous GPER blocking. Data were expressed as mean ± SEM (*P < 0.05, **P < 0.01, ***P < 0.001). **a** Representative images of H&E staining in the ileum following 5-FU administration to show the effect of G-1 on the histopathological damage in CIM model (scale bars: 50 μm). **b** The statistical graph of mucosal damage score and crypt damage score within four subgroups to show the effect of GPER activation on histological injury in the CIM model (*n* = 10). At least 20 villi and crypts of each slide were observed randomly to get the score of intestinal mucosa injury and crypt injury. **c** The effect of GPER activation with G-1 on the body weight loss from day 0 to day 5 in 5-FU induced CIM (*n* = 10). The data were expressed as a percentage change compared to pre-treatment. **d** Representative images of H&E staining in the ileum following 5-FU administration to show the effect of G15 treatment alone on the histopathological damage in the CIM model (scale bars: 50 μm). **e** The statistical graph of mucosal damage score and crypt damage score within three subgroups to show the effect of G15 blocking endogenous GPER on the histological damage of CIM (*n* = 6). **f** The effect of endogenous GPER blocking with G15 on the weight loss from day 0 to day 5 in the CIM model (*n* = 6). The data were expressed as a percentage change compared to pre-treatment. **g** Effects of 5-FU on tumor size in tumor-bearing mice with or without G-1. 4 × 10^6^ LL/2 (Lewis lung carcinoma) cells were injected into the dorsal flank of the male C57BL/6 mice to establish the tumor-xenograft model (*n* = 5).

Increased inflammatory infiltration, abruption between the epithelial layer and lamina propria, shortening of villus length, and necrosis of crypt cells was observed in 5-FU-induced CIM model. G-1 reversed the increased intestinal mucosal and crypt damage scores (Fig. 1a, b). Accordingly, the significant body weight loss induced by 5-FU was inhibited by G-1 (Fig. 1c). All of these effects of G-1 were abolished by G15, the selective GPER antagonist (Fig. 1a–c). Moreover, G15 blockade of endogenous GPER activation resulted in more serious body weight loss and intestinal damage in the 5-FU treated group (Fig. 1d–f). The tumor-bearing experiment indicated that the antitumor effect of 5 -FU was not affected by G-1 (Fig. 1g).

### GPER activation protected mucosal barrier function in 5-FU-induced CIM

**Fig. 2:**
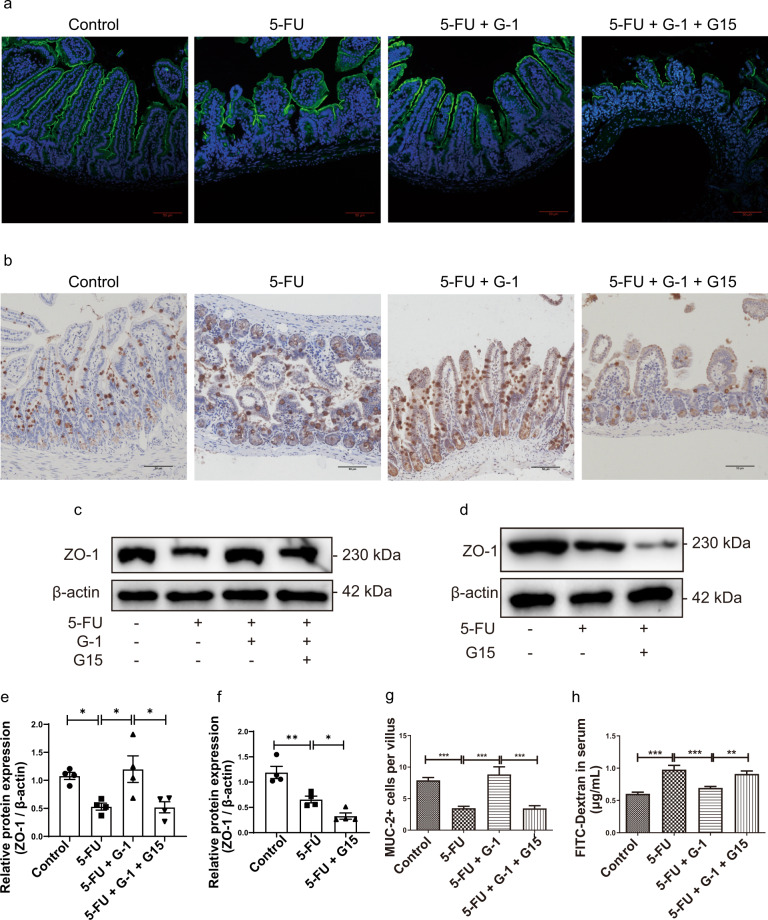
Effect of GPER on the intestinal mucosal barrier in 5-FU induced CIM. CIM model was induced by i.p. injection 5-FU (30 mg/kg/day) for 5 days. G-1 (0.03 mg/kg/day), G15 (0.3 mg/kg/day) were administrated intraperitoneally with 5-FU together. G-1 was administrated alone or with G15 in CIM model to test the effect of GPER activation. G15 was used alone to test the effect of endogenous GPER blocking. On the 5^th^ day, the ileum was collected for immunofluorescence, immunohistochemical staining and western blot. Data were expressed as mean ± SEM (*P < 0.05, **P < 0.01, ***P < 0.001). **a** Immunofluorescence for ZO-1 in ileum to show the effect of G-1 on ZO-1 expression in the CIM model (scale bars: 50 μm). **b** Representative images for immunohistochemical staining of Muc-2 in ileum following G-1 administration with or without G15 in the CIM model (scale bars: 50 μm). **c** Representative western blots photographs for ZO-1 following G-1 administration with or without G15 in the CIM model. **d** Representative western blots photographs for ZO-1 following endogenous GPER blocking with G15 alone in the CIM model. **e** Statistical analysis of ZO-1 expression in ileum tissue within four subgroups to show the effect of GPER activation on ZO-1 expression in the CIM model (*n* = 4). **f** Statistical analysis of ZO-1 expression in ileum tissue within three subgroups to show the effect of endogenous GPER blocking on ZO-1 expression in the CIM model (*n* = 4). **g** Effect of GPER activation on the number of Muc-2^+^ cells per villus within four subgroups in the CIM model. At least 20 villi were counted randomly for each slide, and the mean value was calculated for single sample (*n* = 6). **h** Effect of GPER activation with G-1 on ileal mucosal permeability within four subgroups in the CIM model (*n* = 6).

Destruction of the mucosal barrier is a key pathological change in CIM, so we examined the tight junction protein ZO-1 and goblet cells (cellular sources of the intestinal mucus barrier). ZO-1 distribution was disintegrated and partly absent after 5-FU treatment, and the expression of ZO-1 protein decreased. These abnormalities were reversed by G-1, G15 completely blocked the effects of G-1 (Fig. 2a, c, e). G15 alone aggravated the reduction in ZO-1 expression during CIM (Fig. 2d, f). 5-FU induced mucin-2 (Muc-2) ^+^ goblet cells diminution was regained by G-1 (Fig. 2b, g). Accordingly, G-1 treatment restored the increased mucosal permeability caused by CIM, which was blocked by G15 (Fig. 2h).

### GPER activation inhibited crypt cell apoptosis and protected cell proliferation in the CIM model

G-1 alleviated the weight loss, mucosal and crypt damage, decline in villous height and crypt depth induced by cisplatin (Supplementary Fig. S1), suggesting that G-1 might offer protection from CIM by some general mechanism, not just for the 5-FU. Crypt cell injury is the key mechanism of CIM [4, 8, 34, 35], so we next tested the effects of G-1 on the apoptosis and proliferation of crypt cells in CIM.

**Fig. 3:**
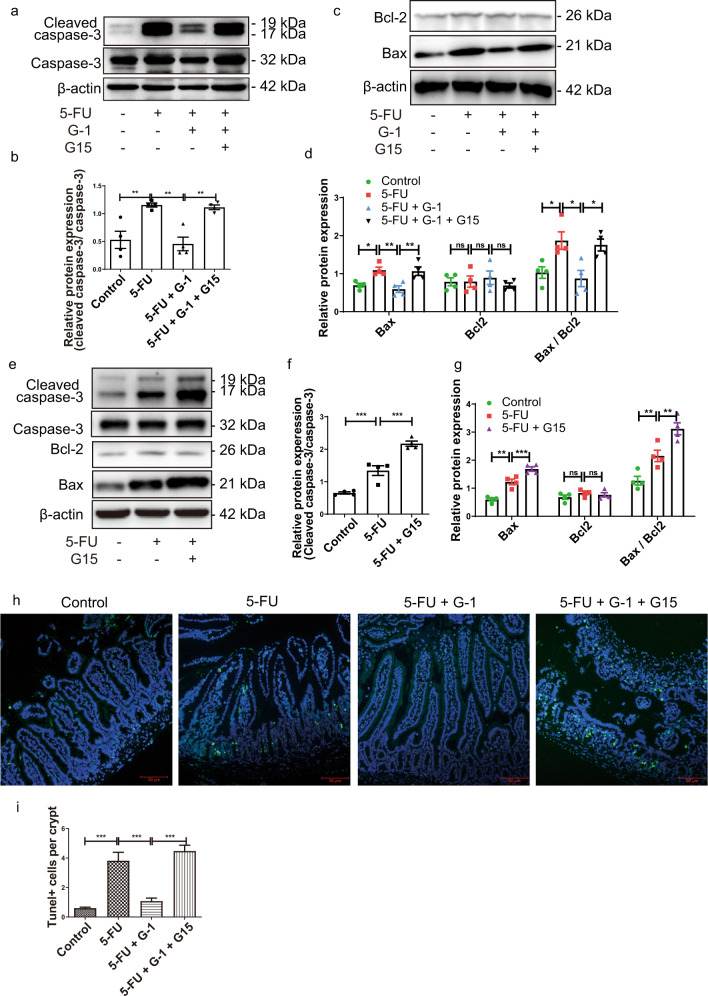
Effect of GPER on the apoptosis of crypt cell in 5-FU induced CIM. CIM model was induced by i.p. injection 5-FU (30 mg/kg/day) for 5 days. G-1 (0.03 mg/kg/day) and/or G15 (0.3 mg/kg/day) were administrated intraperitoneally with 5-FU together. G-1 was administrated alone or with G15 together in CIM model to test the effect of GPER activation. G15 was used alone to test the effect of endogenous GPER blocking. On the 5^th^ day, the ileum was collected for immunofluorescence, immunohistochemical staining and western blot. Data were expressed as mean ± SEM (*P < 0.05, **P < 0.01, ***P < 0.001). **a** Representative western blots photographs for Cleaved caspase-3 and Caspase-3 expression following G-1 administration with or without G15 in the CIM model. **b** Statistical analysis of Cleaved caspase-3 expression in ileum tissue within four subgroups to show the effect of GPER activation on apoptosis in the CIM model (*n* = 4). **c** Representative western blots photographs for Bcl-2 and Bax expression following G-1 administration with or without G15 in the CIM model. **d** Statistical analysis of Bcl-2, Bax expression and Bax/ Bcl-2 ratio within four subgroups to show the effect of GPER activation on apoptosis in the CIM model (*n* = 4). **e** Representative western blots photographs for Cleaved caspase-3, Caspase-3, Bcl-2, Bax expression following G15 administration alone in the CIM model. **f** Statistical analysis of Cleaved caspase-3 expression in ileum tissue within three subgroups to show the effect of endogenous GPER blocking with G15 on apoptosis in the CIM model (*n* = 4). **g** Statistical analysis of Bcl-2, Bax expression and Bax/ Bcl-2 ratio in ileum tissue within three subgroups to show the effect of endogenous GPER blocking with G15 on apoptosis in the CIM model (*n* = 4). **h** Representative images for TUNEL staining in ileum following G-1 administration with or without G15 in the CIM model (scale bars: 50 μm). **i** Statistical graph for the number of TUNEL + cells per crypt within four subgroups to show the effect of GPER activation on apoptosis of crypt cell in the CIM model. At least 20 crypts were counted randomly for each sample, and the mean value was calculated for single sample (*n* = 4).

Treatment with 5-FU induced the upregulation of cleaved caspase-3 and Bax in ileum, and increased Bax/Bcl-2 ratio, while Bcl-2 expression was stable. The application of G-1 reversed the increase of pro-apoptotic protein expression in CIM, which was blocked by G15 (Fig. 3a–d). G15 alone promoted the upregulation of proapoptotic proteins in the CIM model (Fig. 3e–g). G-1 treatment inhibited the increase in the number of TUNEL^+^ cells in the crypts of the CIM model (Fig. 3h–i), suggesting protective effect on crypt cells.

**Fig. 4:**
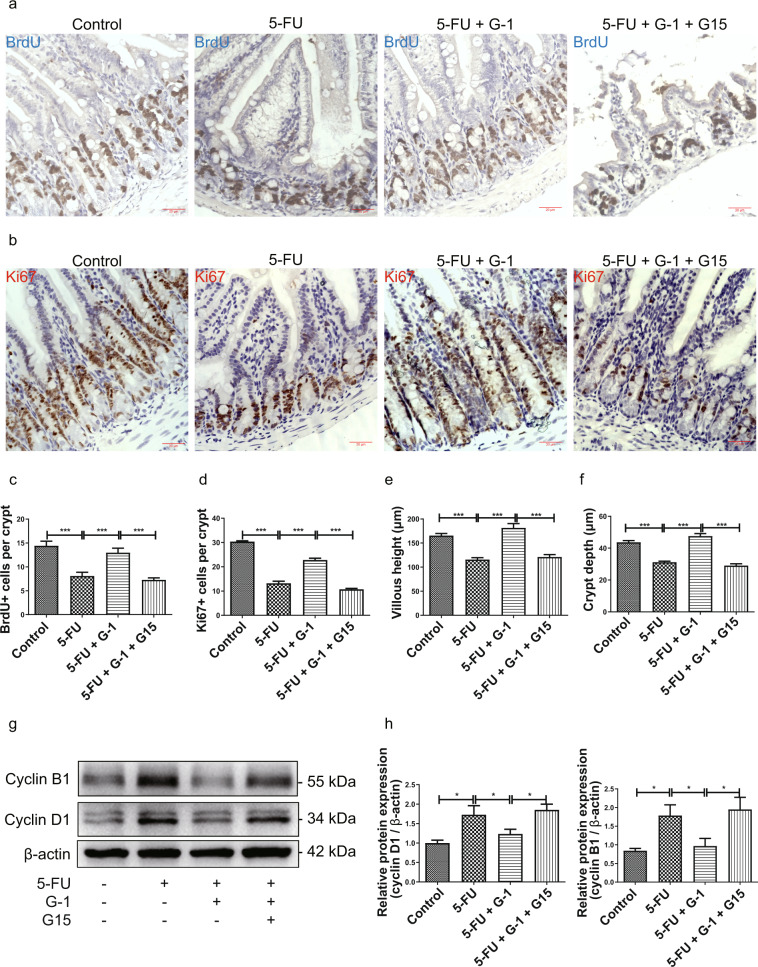
Effect of GPER activation on crypt cell proliferation in 5-FU induced CIM. CIM model was induced by i.p. injection 5-FU (30 mg/kg/day) for 5 days. G-1 (0.03 mg/kg/day) and/or G15 (0.3 mg/kg/day) were administrated intraperitoneally with 5-FU together. G-1 was administrated alone or with G15 together in the CIM model to test the effect of GPER activation. On the 5^th^ day, the ileum was collected for immunohistochemical staining and western blot. Data were expressed as mean ± SEM (*P < 0.05, ***P < 0.001). **a** Representative images for immunohistochemical staining of BrdU following G-1 administration with or without G15 in the CIM model (scale bars: 20 μm). **b** Representative images for immunohistochemical staining of Ki67 following G-1 administration with or without G15 in the CIM model (scale bars: 20 μm). **c** Statistical graph for the number of BrdU^+^ cells per crypt within four subgroups to show the effect of GPER activation on proliferation of crypt cells in the CIM model. At least 20 crypts were counted randomly for each sample, and the mean value was calculated for single sample (*n* = 6). **d** Statistical graph for the number of Ki67^+^ cells per crypt within four subgroups to show the effect of GPER activation on proliferation of crypt cells in the CIM model. At least 20 crypts were counted randomly for each sample, and the mean value was calculated for single sample (*n* = 6). **e** Statistical graph of villous height following G-1 administration with or without G15 in the CIM model. At least 20 villi were counted randomly for each sample, and the mean value was calculated for single sample (*n* = 10). **f** Statistical graph of crypt depth following G-1 administration with or without G15 in the CIM model. At least 20 crypts were counted randomly for each sample, and the mean value was calculated for single sample (*n* = 10). **g** Representative western blots photograph to show the effect of G-1 administration with or without G15 on cyclin D1 and cyclin B1 expression in the CIM model. **h** Statistical analysis of cyclin D1 and cyclin B1expression within four subgroups to show the effect of GPER activation on cyclin D1 and cyclin B1 expression in the CIM model (*n* = 5).

Our previous studies showed that GPER in jejunum crypts had a protective effect on the proliferation of crypt cells after injury [28]. Here, we found the reduction of BrdU^+^ (marking S phase cells) and Ki67^+^ cells (marking proliferating cells) in the crypts induced by 5-FU was corrected by G-1 treatment (Fig. 4a,d). Morphologically, the reductions in villous height and crypt depth were restored by G-1 (Fig. 4e, f). G15 abolished these beneficial effects of G-1 on crypt cell in CIM (Fig. 4a–f). Cyclins are the key regulators of cell cycle, so we examined the expression of cyclin D1 and B1. These cyclins were highly expressed in the 5-FU treated-group, while GPER activation reversed their abnormal expression (Fig. 4g, h).

### G-1 treatment prevented DNA damage in crypt cells in the CIM model

**Fig. 5:**
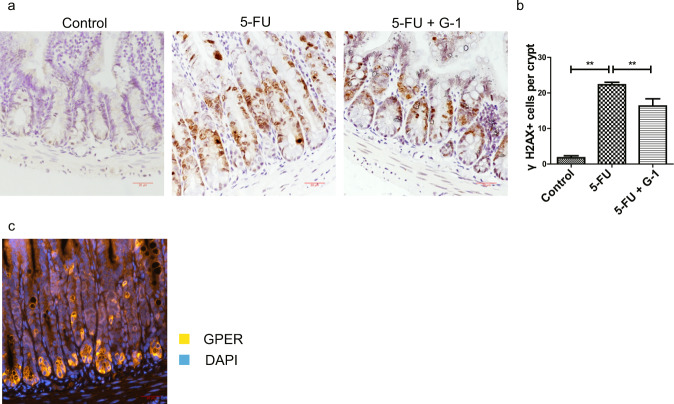
Effect of G-1 treatment on DNA damage of crypt cells induced by 5-FU. CIM model was induced by i.p. injection 5-FU (30 mg/kg/day) for 5 days. G-1 (0.03 mg/kg/day) were administrated intraperitoneally with 5-FU together. **a** Representative immunohistochemical staining for γH2AX in ileum for 5-FU group with or without G-1 administration and for control group (Scale bars: 20 μm). **b** Statistical graph of γH2AX^+^ cells in ileal crypt within three subgroups to show the effect of GPER activation on DNA damage of crypt cells induced by 5-FU. 15 crypts were randomly calculated in each section, and the average value was obtained. Data were expressed as mean ± SEM (*n* = 6, **P < 0.05). **c** Immunofluorescence staining of GPER in ileal crypts in C57BL/6 mice (Scale bar: 20 μm).

DNA damage is the key mechanism by which antineoplastic agents induce cell apoptosis and decrease cell proliferation [15]. γH2AX is the phosphorylated form of histone H2AX, a special DNA damage marker [36]. After G-1 treatment, 5-FU-induced γH2AX^+^ cell increasing was significantly reduced, indicating DNA damage inhibition (Fig. 5a, b). GPER was predominantly expressed in ileum crypts (Fig. 5c), in line with its effect on DNA damage in crypt cells.

### GPER activation restored the ERK1/2 activity of crypt cells in the CIM model

**Fig. 6:**
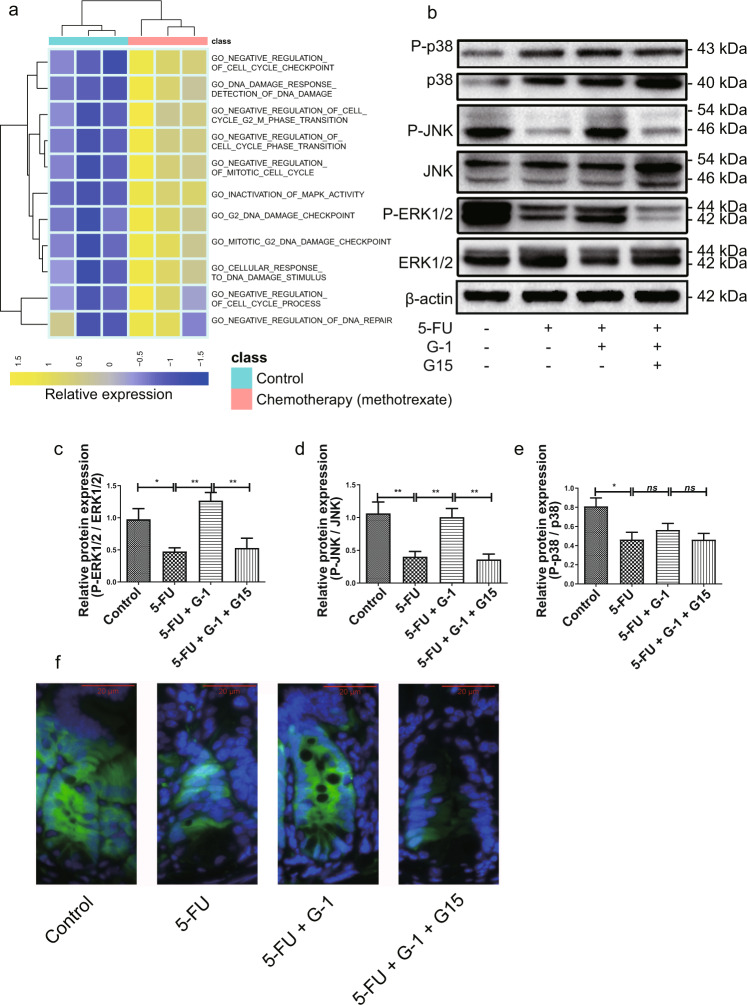
Effect of GPER activation on ERK1/2 activity in the CIM model. CIM model was induced by i.p. injection 5-FU (30 mg/kg/day) for 5 days. G-1 (0.03 mg/kg/day) and/or G15 (0.3 mg/kg/day) were administrated intraperitoneally with 5-FU together. G-1 was administrated alone or with G15 together in the CIM model to test the effect of GPER activation. On the 5^th^ day, the ileum was collected for immunofluorescence staining and western blot. Data were expressed as mean ± SEM (*P < 0.05, **P < 0.01). **a** The GSVA analysis showed that MAPK signaling pathway was inactivated under the chemotherapeutic drug methotrexate treatment, with the enhancement of DNA damage and cell cycle arrest. **b** Representative western blots photographs for MAPK signal pathway from the control group and from the 5-FU treated group with or without G-1, G15 application. **c** Statistical analysis of P-ERK1/2 within four subgroups to show the effect of GPER activation on the activity of ERK1/2 in the CIM model (*n* = 5). **d** Statistical analysis of P-JNK within four subgroups to show the effect of GPER activation on the activity of JNK in the CIM model (*n* = 5). **e** Statistical analysis of P-p38 within four subgroups to show the effect of GPER activation on the activity of p38 in the CIM model (*n* = 5). **f** Immunofluorescence staining for P-ERK1/2 in ileum from the control group and from the 5-FU treated group with or without G-1, G15 application (Scale bars: 20 μm).

Gene expression data from intestinal tissue after chemotherapy were found in the GEO and the matrix was downloaded. We performed GSVA to visualize the biological processes involved in the biological function alterations in intestinal tissue after chemotherapeutic drug methotrexate treatment, which showed that the MAPK signals were inactivated, cell cycle arrest and DNA damage were enhanced (Fig. 6a). Based on these data, we examined whether GPER activation affected the MAPK signaling pathway in CIM. The MAPK signaling pathway was significantly inhibited by 5-FU treatment, and the activities of ERK1/2, JNK and p38 were downregulated. G-1 treatment reactivated P-ERK1/2 and P-JNK in the CIM model, while it exerted no effect on p38 phosphorylation (Fig. 6b–e). The effect of G-1 on ERK1/2 activity was blocked by G15 (Fig. 6b–e). Moreover, the basal P-ERK1/2 activity of C57BL/6 mice increased after 5 days of G-1 treatment, suggesting that the effect of G-1 on ERK1/2 activity of CIM might be independent of 5-FU (Supplementary Fig. S2). Immunofluorescence staining showed that G-1-induced recovery of P-ERK1/2 expressions was located in the crypts in the CIM model (Fig. 6f), while P-JNK was mainly located in the mesenchymal area of villi (data not shown). So considering the reported association between ERK1/2 activity and DNA damage [37] we theorized that recovery of ERK1/2 activity in crypts might be the key mechanism by which GPER activation inhibits DNA damage in CIM.

### GPER activation inhibited 5-FU induced DNA damage in cultured IEC-6 cells by restoring ERK1/2 activity

**Fig. 7:**
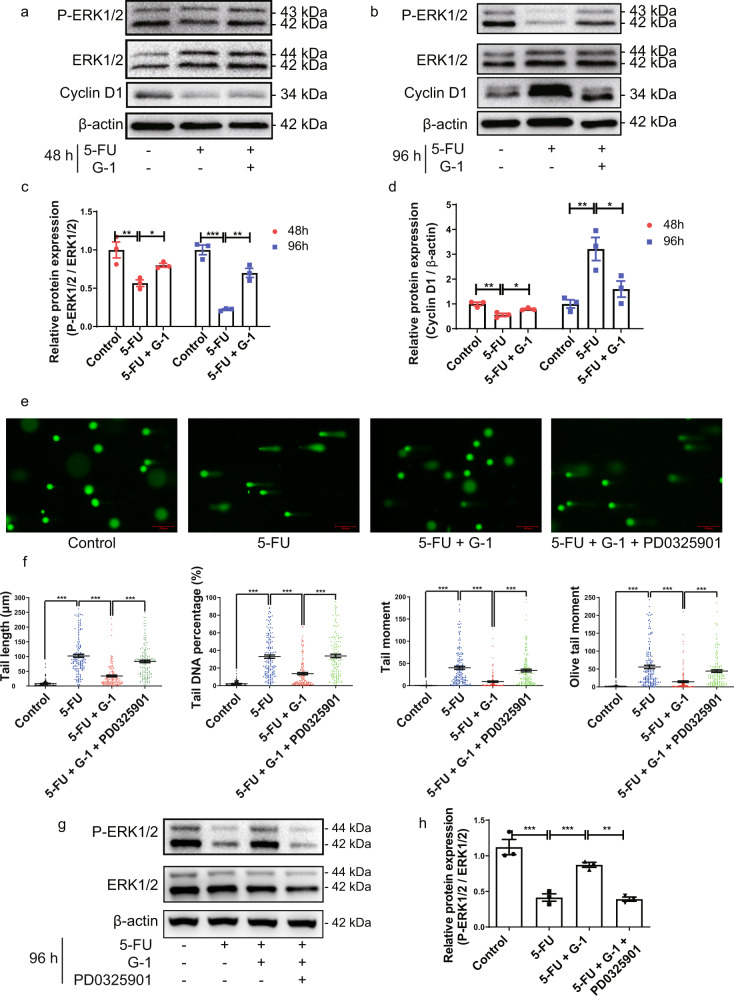
Role of ERK1 / 2 activity in GPER protection against 5-FU induced DNA damage in IEC-6 cells. IEC-6 cells were stimulated under 10^−4^ mol/L 5-FU for 48 h or 96 h with or without G-1 application (10^−7 ^mol/L). The selective ERK1/2 inhibitor PD0325901(5 × 10^−^^3^ mol/L) was administrated to block the ERK1/2 phosphorylation. Data were expressed as mean ± SEM (*P < 0.05, **P < 0.01, ***P < 0.001). **a** Representative western blots photographs for P-ERK1/2 and cyclin D1 in cultured IEC-6 cells exposure to 5-FU for 48 h in the presence or absence of G-1. **b** Representative western blots photographs for P-ERK1/2 and cyclin D1 in cultured IEC-6 cells exposure to 5-FU for 96 h in the presence or absence of G-1. **c** Statistical analysis of P-ERK1/2 expressions in IEC-6 cells exposed to 5-FU for 48 h or 96 h in the presence or absence of G-1 (*n* = 3). **d** Statistical analysis of cyclin D1 expressions in IEC-6 cells exposed to 5-FU for 48 h or 96 h in the presence or absence of G-1 (*n* = 3). **e** Representative images of comet assay for IEC-6 cells showing the effect of PD0325901 on G-1 inhibition of 5-FU induced DNA damage (scale bars: 100 μm). **f** Statistical graph of comet assay. Tail length, tail DNA percentage, tail moment and olive tail moment of IEC-6 within four subgroups to show the effect of PD0325901 on G-1 inhibition of 5-FU induced DNA damage. The experiment was repeated three times independently, 50 cells of each time were analyzed (*n* = 150). **g** Effect of PD0325901 on G-1 protection of P-ERK1/2 activities in 5-FU treated cells (*n* = 3).

With cultured IEC-6 cells we investigated the causal relationship between GPER protection of ERK1/2 activity and inhibition of DNA damage following exposure to 5-FU. ERK1/2 activity was inhibited in a time-dependent manner after 5-FU stimulation for both 48 h and 96 h, G-1 treatment restored ERK1/2 activity and the effect at 96 h was more obvious than that at 48 h (Fig. 7a–c). The expression of cyclin D1 decreased 48 h after 5-FU stimulation but increased significantly at 96 h, and both effects were inhibited by G-1 treatment (Fig. 7a, b, d). G-1 treatment inhibited the increase in tail length, tail DNA percentage, tail moment and olive tail moment as evaluated by comet assay after 5-FU treatment for 96 h (Fig. 7e, f). Notably, the G-1-induced DNA damage inhibition disappeared after the application of the ERK1/2 inhibitor PD0325901, which eliminated the effects of G-1 on ERK1/2 activity (Fig. 7e–g). This is direct evidence of a causal regulatory relationship by GPER on ERK1/2 activity and DNA damage inhibition.

### The effect of GPER activation on 5-FU induced CIM was inhibited by ERK1/2 inhibitor in vivo

**Fig. 8:**
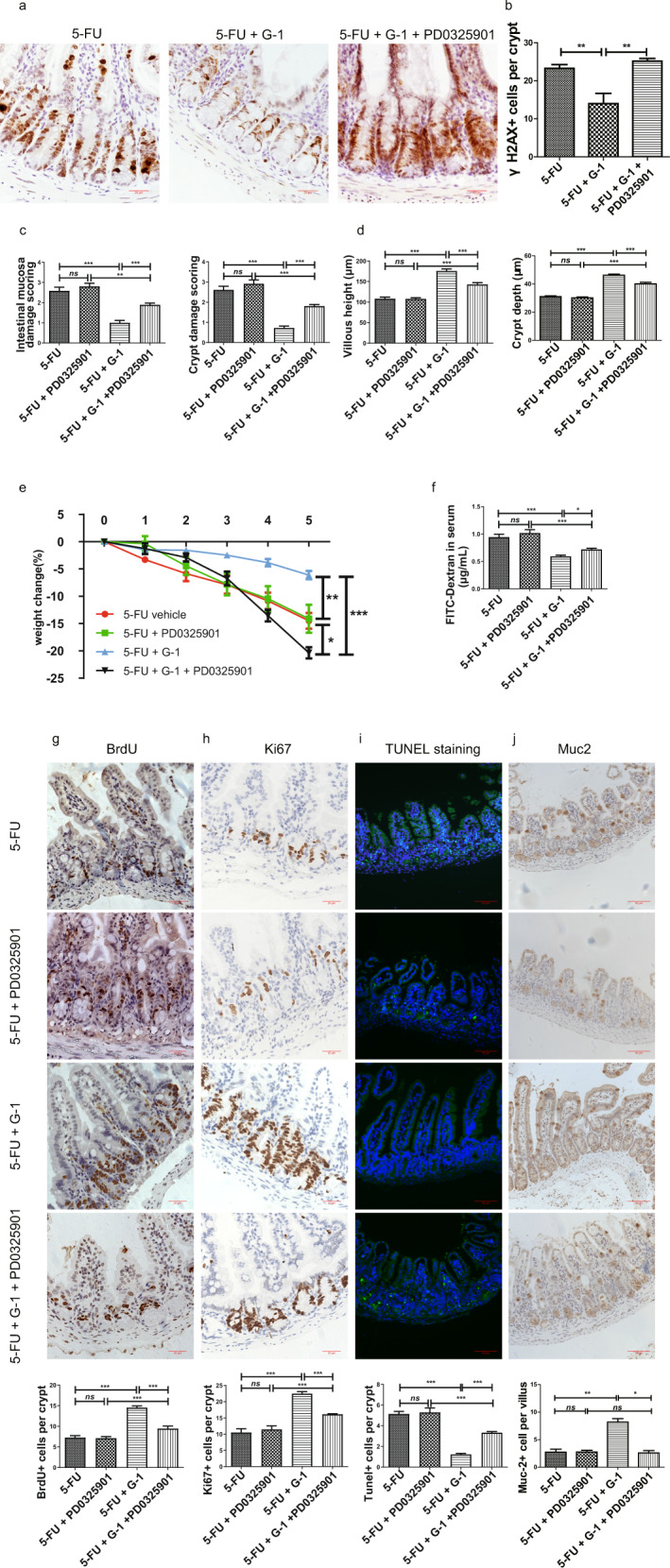
The role of regulating ERK1/2 activity in GPER activation protecting 5-FU induced CIM. CIM model was induced by i.p. injection 5-FU (30 mg/kg/day) from day 0 to day 5. G-1 (0.03 mg/kg/day) and/or ERK1/2 inhibitor PD0325901 (5 mg/kg/day) were administrated intraperitoneally together with 5-FU. On the 5^th^ day, the ileum was collected for H& E staining, immunofluorescence staining, immunohistochemical staining and western blot. Data were expressed as mean ± SEM (*P < 0.05, **P < 0.01, ***P < 0.001). **a** Representative immunohistochemical images of γH2AX staining in the ileum of the CIM model following G-1 administration or in combination with PD0325901 (Scale bars: 20 μm). **b** Statistical graph of γH2AX^+^ cells in ileal crypt within three subgroups to show the role of regulating ERK1/2 activity in GPER inhibiting DNA damage induced by CIM. 15 crypts were randomly calculated in each section, and the average value was obtained (*n* = 5). **c** Effect of ERK1/2 inhibitor PD0325901 on G-1 in reducing intestinal mucosal damage score and crypt damage score of CIM model (*n* = 9). **d** Effect of ERK1/2 inhibitor PD0325901 on G-1 inhibition of crypt depth reduction and villus shortening in the CIM model (*n* = 9). At least 20 villi were counted randomly for each sample, and the mean value was calculated for single sample. **e** Effect of ERK1/2 inhibitor PD0325901 on G-1 inhibition of weight loss in the CIM model (*n* = 9). **f** Effect of ERK1/2 inhibitor PD0325901on G-1 protected ileal mucosal permeability in the CIM model (*n* = 6). The mucosal permeability was evaluated by the concentration of Fluorescein isothiocyanate (FITC) -dextran in blood. **g** Representative immunohistochemical images of BrdU staining within four subgroups (scale bars: 20 μm) and statistical graph of BrdU^+^ cell to show the effect of ERK1/2 inhibitor PD0325901 on G-1 protecting proliferation of crypt cells in the CIM model. At least 20 crypts were counted randomly, and the mean value was calculated for single sample (*n* = 6). **h** Representative immunohistochemical images of Ki67 staining within four subgroups (scale bars: 20 μm) and statistical graph of Ki67^+^ cell to show the effect of ERK1/2 inhibitor PD0325901 on G-1 protecting proliferation of crypt cells in the CIM model. At least 20 crypts were counted randomly, and the mean value was calculated for single sample (*n* = 6). **i** Representative images of TUNEL staining within four subgroups (scale bars: 50 μm) and statistical graph of TUNEL ^+^ cell to show the effect of ERK1/2 inhibitor PD0325901 on G-1 inhibiting apoptosis of crypt cells in the CIM model. At least 20 crypts were counted randomly, and the mean value was calculated for single sample (*n* = 6). **j** Representative immunohistochemical images of Muc-2 staining within four subgroups (scale bars: 50 μm) and statistical graph of Muc-2^+^ cells to show the effect of ERK1/2 inhibitor PD0325901 on G-1 protecting the mucus barrier in the CIM model. At least 20 villi were counted randomly, and the mean value was calculated for single sample (*n* = 6).

To compensate the limitations of the in vitro experiment, we used in vivo experiments to verify the central role of protecting ERK1/2 activity in G-1 protection against CIM. In vivo application of PD0325901 blocked the effects of G-1 on ERK1/2 activity in the CIM model (Supplementary Fig. S3). Along with the loss of P-ERK1/2 protections, the inhibitory effects of G-1 on DNA damage in crypt cells were abolished (Fig. 8a, b). PD0325901 inhibited all of the indicators that G-1 protected against CIM, including weight loss inhibition, tissue injury and mucosal permeability improvement, reversal of BrdU^+^ and Ki67^+^ cell reduction and TUNEL^+^ cell increase in crypts, as well as the recovery of cyclin D1 and cyclin B1 expression (Fig. 8c–i, Supplementary Fig. S4). It was puzzling that the G-1 and PD0325901 combination treatment caused the weight loss to be more serious in the CIM model (Fig. 8e), this effect might be related to other mechanisms that were not studied here. In addition, PD0325901 completely blocked the effect of G-1 treatment on the number of Muc-2^+^ cells in the CIM model (Fig. 8j). PD0325901 alone did not further exaggerated the inhibition of p-ERK1/2 activities in CIM, so did the abnormal performances in CIM (Fig. S3, Fig. 8c–j).

## Discussion

CIM has severe negative impacts on the prognosis and lifespan of patients during anti-tumor treatment [1], but limited effective options are available to clinicians and patients to prevent or relieve the syndromes [38]. Our study revealed that GPER activation reduced the intestinal histological damage, weight loss, and mucosal barrier dysfunction, protected crypt cell proliferation and inhibited apoptosis in a CIM model. These effects of GPER activation on CIM were partly achieved by restoring ERK1/2 activity, thereby inhibiting DNA damage in crypt cells.

The rapid proliferation of intestinal epithelial cells, coupled with complex immune effects and interactions with the intestinal microbiota, makes the gastrointestinal tract particularly vulnerable to antineoplastic agents [39]. 5-FU is a classical chemotherapeutic agent [40], its use to induce CIM has been widely adopted in research. With this model we first demonstrated GPER activation protected CIM by targeting crypt cells. GPER activation significantly diminished the manifestations of CIM, such as weight loss, damage to the intestinal mucosa and crypt, alterations to the expression and distribution of ZO-1, and the reduction in the number of goblet cells. Tight junction proteins connecting adjacent IECs and mucosal components secreted by goblet cells are essential to maintain the mucosal barrier [41]. Therefore, G-1 treatment abolished the increase in intestinal mucosal permeability in the CIM model. Similar effects on mucosal permeability after G-1 treatment have been reported in other pathological models, such as intestinal ischemia-reperfusion injury [28], global cerebral ischemia [42] and α-hemolysin-mediated disruption of epithelial barrier integrity [43]. All of the beneficial effects of G-1 were blocked by the selective GPER antagonist, G15. Moreover, the protection of GPER against CIM was not 5-FU specific because G-1 also protected cisplatin-induced CIM.

Chemotherapeutic drugs such as 5-FU, cisplatin and doxorubicin cause CIM by inducing crypt cell apoptosis and inhibiting cell proliferation [8, 34, 35]. In our CIM model, the expression of the proapoptotic proteins cleaved caspase-3, Bax and the Bax/Bcl-2 ratio was upregulated while Bcl-2 expression remained unchanged, which was consistent with previous reports [44, 45]. Upregulation of cleaved caspase-3, Bax and Bax/Bcl-2 ratio was reversed by G-1, suggesting increased enterocyte survival. TUNEL staining showed that the decrease in the number of apoptotic cells in the CIM model following G-1 treatment were mainly distributed in the intestinal crypts, indicating G-1 inhibited the toxic effects of chemotherapy on crypt cells. Consistent with this result, G-1 treatment improved the proliferation of crypt cells in the CIM model, as confirmed by the increase in the number of BrdU^+^ and Ki67^+^ cells within the crypts. Accordingly, the decreases in the villus height and crypt depth in CIM model was restored by GPER activation. G15 abolished the effects of G-1 on apoptosis and proliferation of crypt cells. Following blocking endogenous GPER activation with G15, 5-FU-induced apoptosis was significantly aggravated, and increasing of injury as observed by certain parameters, such as weight loss and histological damage, further suggesting the role of GPER in CIM. This inhibition of apoptosis and improvement in proliferation might be the key reasons why GPER inhibits intestinal mucositis.

The levels of cyclins and their spatiotemporal localization have been suggested as crucial determinants of cell fate. Cyclin D1 and B1 promote the transformation of G1 → S phase and G2 → M phase in the cell cycle respectively. Decreasing the expression of cyclin D1 and B1 was related with the dysfunction of cell cycle transition and proliferation inhibition, like the BrdU^+^ cells decreasing [46, 47]. However, here, the decrease in the numbers of BrdU^+^ and Ki67^+^ cells following 5-FU treatment was accompanied by a significant increase in the expression of both cyclin D1 and cyclin B1. 5-FU has been corroborated to generate mimics uracil incorporated into replicating DNA and inhibiting its extension [48]. Anti-neoplastic agents exert their cytotoxic effects by triggering DNA damage following activation of the DNA damage response (DDR), thus blocking cell cycle progression to repair damaged DNA or induce cell apoptosis [7]. A DNA damage-induced reduction in cyclin D1 expression was associated with the rapid cytoplasmic degradation of cyclin D1 via ubiquitin-mediated proteolysis, leading to early cell cycle arrest in G1 phase, which was necessary for effective repair of DNA damage [49, 50]. On the other hand, chemotherapy-induced cyclin D1 overexpression might be associated with its nuclear accumulation, in which cyclin D1 was recruited to the increasing DNA damage sites for stabilization, thus avoiding proteolysis in the cytoplasm [49–51]. Overexpression of cyclin D1 interferes with normal intra-S phase progression, impairs cell survival [49, 52, 53], and prevents DNA repair [54]. High expression of cyclin B1 has been shown to contribute to cell cycle arrest triggered by DNA double strand breaking after ^125^I treatment [55]. γH2AX is a special DNA damage marker, that recruits DNA damage response factors to mediate apoptosis and suppress proliferation [36]. There was a significant increase in the number of γH2AX^+^ cells in crypts in CIM model, which was reversed by G-1 treatment, suggesting an inhibitory effect of G-1 on enhanced DNA damage. The comet assay is an effective method to analyze the degree of DNA damage in single cell in vitro. With this method, we found that 96 h following 5-FU stimulation, DNA damage in IEC-6 cells was triggered; however, G-1 inhibited this DNA damage and to enhance genomic stability. Therefore, we suspected that the upregulation of cyclin D1 and cyclin B1 in 5-FU treated group suggested the observed apoptosis and inhibition of proliferation were related to DNA damage. Both in vivo and in vitro, the upregulation of cyclin D1 induced by 5-FU was restored by G-1, which should be due to the alleviation of DNA damage [49–51]. These results demonstrated for the first time that GPER activation reduced DNA breakage in crypt cells in CIM model. The prevalent expression of GPER in crypts further strengthened this idea.

We employed GSVA analysis from the GEO database to explore the biological functional alterations to intestinal tissue that may involve GPER during chemotherapy and found that the MAPK signaling pathway was inactivated after methotrexate treatment, with observed enhancements in DNA damage and cell cycle arrest. We suspected that MAPK might be involved in 5-FU-induced CIM and verified it by western blot and immunofluorescence. Three components of the MAPK signaling (ERK1/2, p38 and JNK) were inhibited following exposure to 5-FU. Dephosphorylation of both ERK1/2 and JNK in the CIM model was restored by GPER activation, while G-1 exerted no effect on the activity of p38. Immunofluorescence staining showed that the improvement of P-ERK1/2 expressions in the CIM model after GPER activation was located in intestinal crypts, which was identical to the distribution of GPER and its effects on DNA damage in crypts, suggesting a possible role of ERK1/2 activity in GPER action. Since P-JNK was located in the intestinal villous stroma rather than the intestinal crypts, we did not further study the role of JNK in GPER protection of crypt cells in this study.

Similar to reports in other tissues [20], We found that G-1 treatment activated P-ERK1/2 directly in C57BL/6 mice, suggesting that the recovery of ERK1/2 activity after G-1 treatment might not be 5-FU-specific in the CIM model. This result was consistent with the protective effect of G-1 not only on 5-FU but also on cisplatin-induced CIM, and further indicated the role of ERK1/2. ERK1/2 plays a significant role in DNA damage repair caused by chemotherapy or radiotherapy [56]. ERK1/2 is involved in the DNA damage repair systems via different mechanisms, such as activating poly (ADP-ribose) polymerase-1 (PARP-1) to trigger single stranded break repair [57], promoting YB-1 gene transcription to participate in mismatch repair (MMR) [58], and performing double strand breaking repair (DSBR) via PKCs pathway [59, 60]. Tumors with increased ERK1/2 activity possess a stronger capacity to repair DNA damage and resist chemotherapy or radiotherapy, and the application of ERK1/2 inhibitors sensitizes tumors to chemotherapy or radiotherapy by inducing enhanced DNA damage [61–63]. In the intestine, activation of ERK1/2 was found to be involved in apoptosis resistance induced by DNA damage after radiation [64]. We used IEC-6 cells in vitro to investigate the correlation between GPER’s inhibition of DNA damage and its regulation of ERK1/2. Similar to the in vivo results, 5-FU-induced inhibition of ERK1/2 activity and DAN damage in IEC-6 cells were suppressed by G-1. More importantly, the inhibitory effect of G-1 on DNA damage induced by 5-FU was fully abolished by ERK1/2 inhibitor PD0325901, demonstrating that the protective effects of G-1 on 5-FU induced DNA damage were dependent on the ERK1/2 signaling.

The disadvantage of in vitro experiments was that they cannot completely mimic in vivo conditions, so we also performed in vivo experiments to validate the role of ERK1/2 during GPER protection against CIM. In vivo administration of PD0325901 completely blocked the effect of G-1 on ERK1/2 activity in CIM model, thus eliminating the protective effects of G-1 on DNA damage. Accordingly, PD0325901 inhibited all of the GPER activation-induced protections against CIM, such as histological changes, body weight changes, mucosal permeability, and the proliferation and apoptosis of crypt cells. Slightly different, PD0325901 completely abolished the effects of G-1 on number of goblet cells in CIM model. PD0325901administration partially eliminated the effects of GPER on CIM, suggesting that the protective effects of GPER activation were partly attributable to ERK1/2 and DNA damage. Other mechanisms are also involved in this process, which should be explored in the future. For example, our previous studies have shown GPER activation protected crypt cell proliferation by inhibiting iNOS expression or endoplasmic reticulum stress [28, 29]. PD0325901 alone did not affect ERK1/2 activity in CIM, so did the intestinal damage, suggesting the correlation between ERK1/2 activity and CIM.

In conclusion, we confirmed that GPER activation protects crypt cells in the CIM by ameliorating DNA damage via regulating ERK1/2 activity, thus protecting the proliferation and reducing the apoptosis of crypt cells to ameliorate CIM [65]. Moreover, tumor-bearing experiment mice indicated G-1 application did not alter the anti-tumor effect of 5-FU. The divergent effects of GPER on tumor and crypt cells suggests that GPER could be a useful candidate target to prevent CIM in cancer patients.

## Supplementary information

Supplementary figure legends

Figure S1

Figure S2

Figure S3

Figure S4

## Data Availability

The data used to support the findings of this study are available from the corresponding author upon reasonable request.
