# Supplementary material for: Activation of G protein coupled estrogen receptor prevents chemotherapy-induced intestinal mucositis by inhibiting the DNA damage in crypt cell in an extracellular signal-regulated kinase 1- and 2- dependent manner
Source: Cell Death Dis. 2021 Oct 30;12(11):1034. doi: 10.1038/s41419-021-04325-z (PMC8557214; doi:10.1038/s41419-021-04325-z)
Supplement: Supplementary file 1 — Supplementary figure legends [file 41419_2021_4325_MOESM1_ESM.docx]

**Supplementary figure legends**

Figure S1 Effect of GPER activation on cisplatin induced CIM

Cisplatin (5mg/kg) was injected intraperitoneally on day 0 and day 2. G-1 (0.03 mg/kg/day) was injected intraperitoneally for 5 consecutive days. The weight was monitored from day 0 to day 5 and expressed as a percentage change compared to pre-treatment. On the 5^th^ day, mice were sacrificed and the ileum was collected for H&E staining. Data were expressed as mean±SEM (******P* < 0.05, *******P* < 0.01, ********P* < 0.001）

1. Representative images of intestinal histology of ileum on the 5th day following cisplatin administration to show the effect of G-1 on the histopathological damage in cisplatin induced CIM (scale bars: 50 μm).
2. The statistical graph of mucosal damage score following G-1 administration in cisplatin induced CIM (n=6). At least 20 villi of each slide were observed randomly to get the score of intestinal mucosa injury.
3. The statistical graph of crypt damage score following G-1 administration in cisplatin induced CIM (n=6). At least 20 crypts of each slide were observed randomly to get the score of crypt injury.
4. The statistical graph of villous height following G-1 administration in cisplatin induced CIM (n=6). At least 20 villi were counted randomly for each sample, and the mean value was calculated for single sample.
5. The Statistical graph of crypt depth following G-1 administration in cisplatin induced CIM (n=6). At least 20 crypts were counted randomly for each sample, and the mean value was calculated for single sample.
6. The effect of GPER activation with G-1 on the body weight loss from day 0 to day 5 in cisplatin induced CIM (n=6).

Figure S2 Effect of G-1 treatment on the expression of P-ERK1/2 in C57BL/6 mice

G-1 (0.03 mg/kg/day) was injected intraperitoneally for 5 consecutive days. The control group was intraperitoneally injected with the same amount of saline.

1. Representative western blots photographs for P-ERK1/2 within two subgroups.
2. Densitometry analysis of P-ERK1/2 to show the effect of G-1 on ERK1/2 activity in C57BL/6 mice (n = 4, **P < 0.05)

Figure S3 Effect of PD0325901 on P-ERK1/2 expressions in G-1 protective CIM model

CIM model was induced by i.p. injection 5-FU (30mg/kg/day) for 5 days. G-1 (0.03 mg/kg/day) and/or PD0325901(5 mg/kg/day) were administrated intraperitoneally with 5-FU together.

1. Representative western blots photographs for P-ERK1/2 within five subgroups.
2. Densitometry analysis of P-ERK1/2 to show the effect of PD0325901 on G-1 regulating ERK1/2 activity in the CIM model (*n* = 4, ******P* < 0.05, ***P*<0.01, ********P* < 0.001）.

Figure S4 The role of regulating ERK1/2 activity in GPER activation protecting 5-FU induced CIM model

CIM model was induced by i.p. injection 5-FU (30mg/kg/day) for 5 days. G-1 (0.03 mg/kg/day) and/or ERK1/2 inhibitor PD0325901(5 mg/kg/day) were administrated intraperitoneally with 5-FU together.

1. Histological representative images in the ileum of the CIM model with G-1 alone or in combination with PD0325901 (scale bars: 50μm).
2. Representative western blots photographs for cyclin D1 and cyclin B1 in the ileum of the CIM model with G-1 alone or in combination with PD0325901.
3. Statistical analysis of cyclin D1 expressions in the ileum of the CIM model with G-1 alone or in combination with PD0325901 (n =4).
4. Statistical analysis of cyclin B1 expressions in the ileum of the CIM model with G-1 alone or in combination with PD0325901 (n =4).
