# Supplementary figures and images for: Activation of G protein coupled estrogen receptor prevents chemotherapy-induced intestinal mucositis by inhibiting the DNA damage in crypt cell in an extracellular signal-regulated kinase 1- and 2- dependent manner
Source: Cell Death Dis. 2021 Oct 30;12(11):1034. doi: 10.1038/s41419-021-04325-z (PMC8557214; doi:10.1038/s41419-021-04325-z)

a

Control

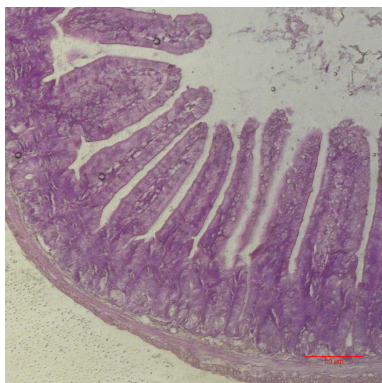

Cisplatin

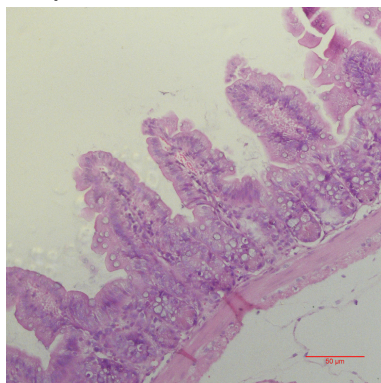

Cisplatin + G-1

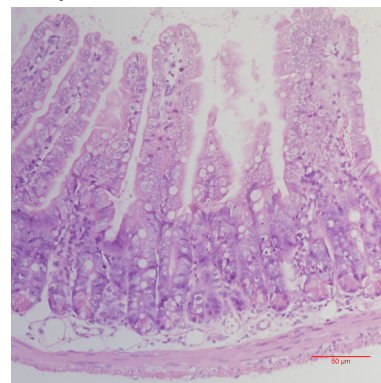

b

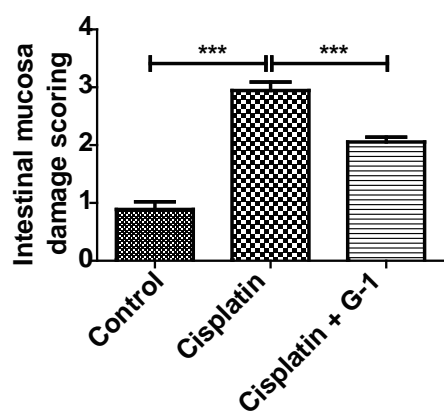

c

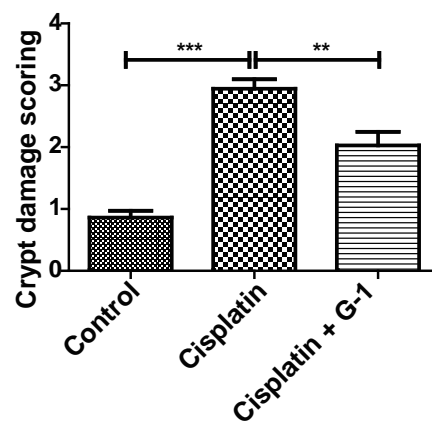

d

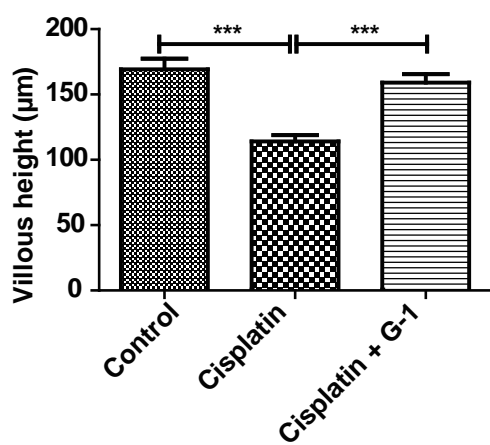

e

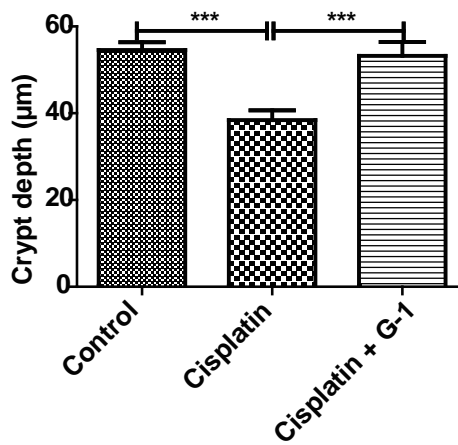

f

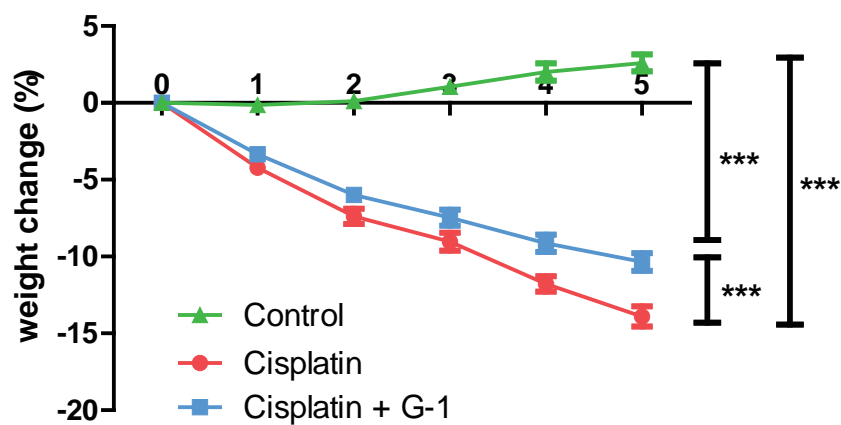

Figure S1

Supplement: Supplementary file 2 — Figure S1 [file 41419_2021_4325_MOESM2_ESM.pdf]

a

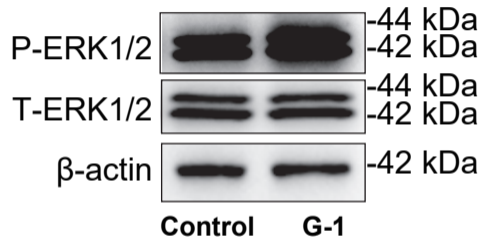

b

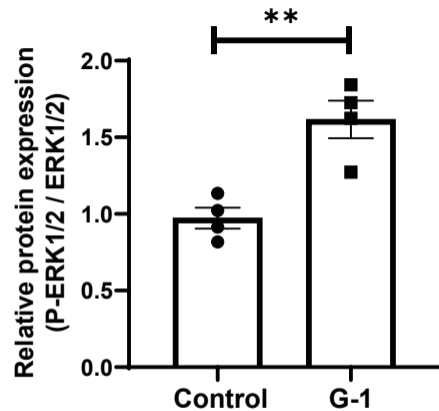

Figure S2

Supplement: Supplementary file 3 — Figure S2 [file 41419_2021_4325_MOESM3_ESM.pdf]

a

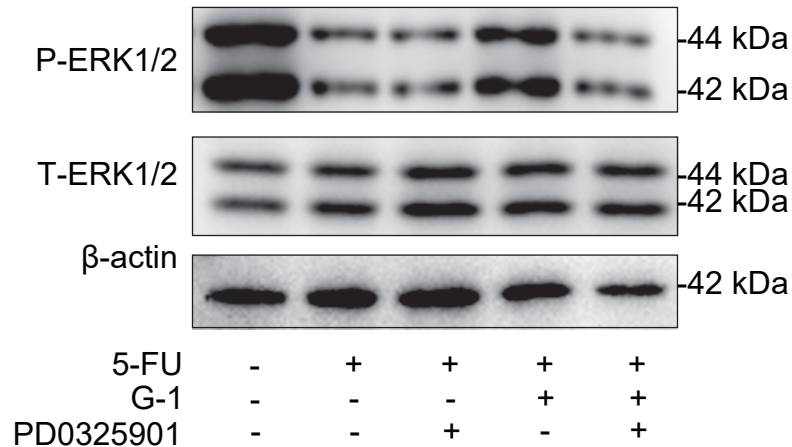

b

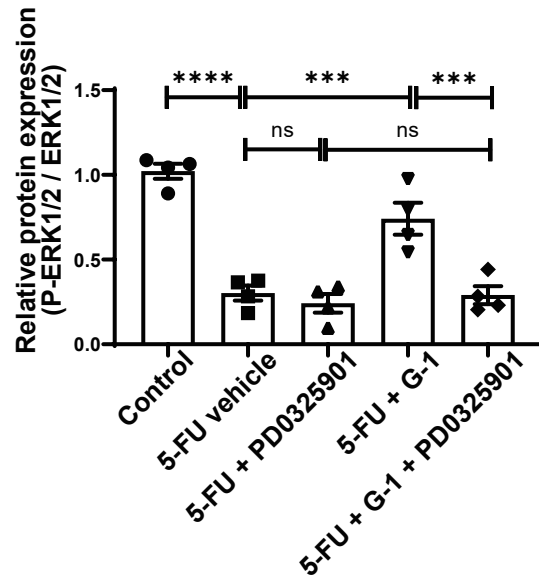

Figure S3

Supplement: Supplementary file 4 — Figure S3 [file 41419_2021_4325_MOESM4_ESM.pdf]

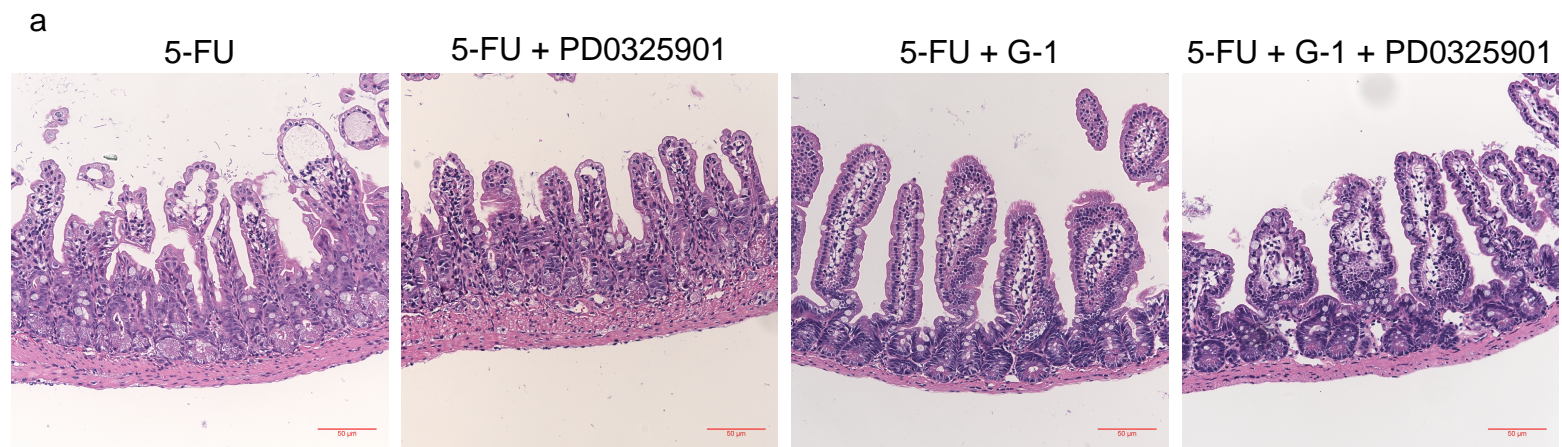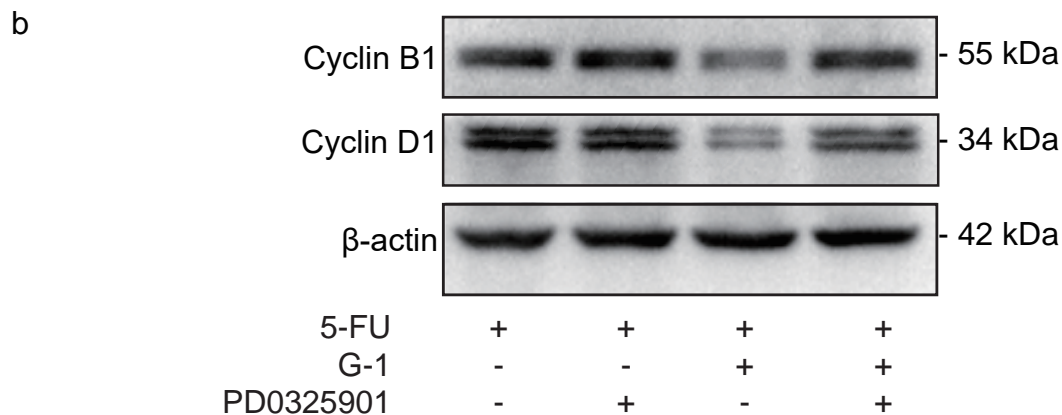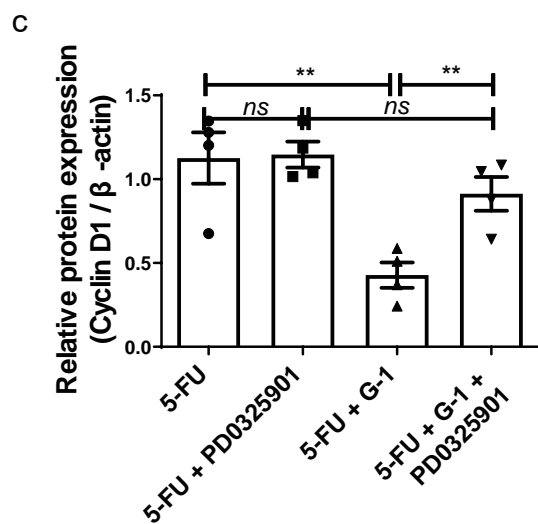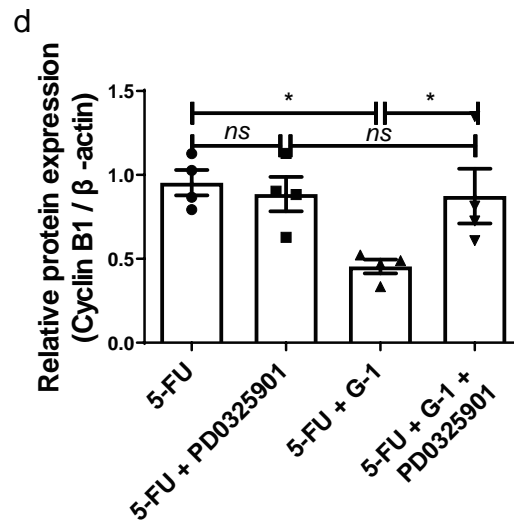

Figure S4

Supplement: Supplementary file 5 — Figure S4 [file 41419_2021_4325_MOESM5_ESM.pdf]
